# Modeling task-specific manifestations of serotonin in basal ganglia using risk-based decision making

**DOI:** 10.1186/1471-2202-15-S1-P83

**Published:** 2014-07-21

**Authors:** B Pragathi Priyadharsini, V Srinivasa Chakravarthy, Balaraman Ravindran, Ahmed A Moustafa

**Affiliations:** 1 Dept. of Biotechnology, Indian Institute of Technology Madras, Chennai 600036, Tamil Nadu, India; 2 Dept. of Computer Science, Indian Institute of Technology Madras, Chennai 600036, Tamil Nadu, India; 3 School of Social Sciences and Psychology, University of Western Sydney, Penrith NSW 2751, Australia

## 

Existing abstract models of serotonin (5HT) in basal ganglia (BG) suffer from their inability to account for the diverse functions of 5HT including punishment prediction and behavioural inhibition, time scale of reward-punishment prediction, and risk sensitivity [1]. We here propose an abstract model of risk based decision making in BG wherein dopamine (DA-denoted by δ) controls the reward prediction error and serotonin (5HT- denoted by α) affects the risk prediction error [1]. This model effectively *reconciles* not only the diverse functions of 5HT but also predicts that BG computes *utility* rather than value, a feature that differentiates from several value-based actor-critic models of BG. Here, value is an expectation of the discounted future rewards, risk is the reward variance, and utility is a weighted summation of the value and risk function with 5HT controlling the weightage or contribution of risk component to utility function. Such a result explains that the primary function of 5HT is to account for the risk computation in decision making—a function that links 5HT to the multiple roles explained above [1]. Just as value has been thought to be computed in the striatum, we propose that utility is also computed in the striatum; and the BG dynamics for utility (instead of value) maximization is described by the Go/Explore/NoGo model of Magdoom et al. (2011)[2]. The proposed abstract model is applied here to a clinical study on the effect of DA agonist medication on reward-punishment sensitivity in Parkinson’s disease (PD) patients (recently / ON, and never / OFF medicated) compared to healthy controls [3]. This experiment demonstrates an increased reward sensitivity in PD-ON, and increased punishment sensitivity in PD-OFF conditions—a trend that is captured by the proposed model of 5HT-DA in BG (Fig. 1A). PD OFF condition is simulated by clamping δ; while PD-ON condition simulation is by multiplying δ with a positive factor (>1).

**Figure 1:**
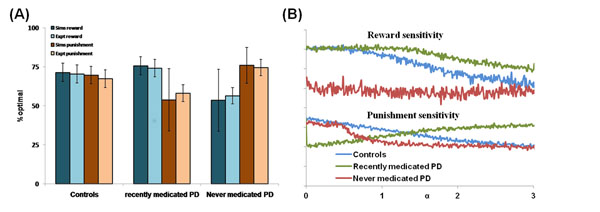
(A) The reward punishment sensitivity for simulated (Sims) PD (α = 0.1) and controls (α = 0.3) to explain the experiment (Expt) [3], (B) Analysis of the effect of 5HT (α) on PD patients' sensitivity profile in comparison to that of controls.

## Conclusion

We propose a significant role of the reduced 5HT levels [4] in addition to DA to accurately explain the observed sensitivity profile [3] (Refer Fig. 1B). Shown here is that the 5HT-DA model (α > 0) captures the experiment profile [3] better than just a DA model of BG (α = 0). This model infers that 5HT along with DA contributes to the PD patients' reward-punishment sensitivity (Fig. 1A,B).
